# A Full Quantum Eigensolver for Quantum Chemistry Simulations

**DOI:** 10.34133/2020/1486935

**Published:** 2020-03-23

**Authors:** Shijie Wei, Hang Li, GuiLu Long

**Affiliations:** ^1^Beijing Academy of Quantum Information Sciences, Beijing 100193, China; ^2^State Key Laboratory of Low-Dimensional Quantum Physics and Department of Physics, Tsinghua University, Beijing 100084, China; ^3^Beijing National Research Center for Information Science and Technology and School of Information Tsinghua University, Beijing 100084, China; ^4^Frontier Science Center for Quantum Information, Beijing 100084, China

## Abstract

Quantum simulation of quantum chemistry is one of the most compelling applications of quantum computing. It is of particular importance in areas ranging from materials science, biochemistry, and condensed matter physics. Here, we propose a full quantum eigensolver (FQE) algorithm to calculate the molecular ground energies and electronic structures using quantum gradient descent. Compared to existing classical-quantum hybrid methods such as variational quantum eigensolver (VQE), our method removes the classical optimizer and performs all the calculations on a quantum computer with faster convergence. The gradient descent iteration depth has a favorable complexity that is logarithmically dependent on the system size and inverse of the precision. Moreover, the FQE can be further simplified by exploiting a perturbation theory for the calculations of intermediate matrix elements and obtaining results with a precision that satisfies the requirement of chemistry application. The full quantum eigensolver can be implemented on a near-term quantum computer. With the rapid development of quantum computing hardware, the FQE provides an efficient and powerful tool to solve quantum chemistry problems.

## 1. Introduction

Quantum chemistry studies chemical systems using quantum mechanics. One primary focus of quantum chemistry is the calculation of molecular energies and electronic structures of a chemical system which determine its chemical properties. Molecular energies and electronic structures are calculated by solving the Schrodinger equation within chemical precision. However, the computational resources needed scale exponentially with the system size on a classical computer, making the calculations in quantum chemistry intractable in high dimension.

Quantum computers, originally envisioned by Benioff, Manin, and Feynman [1–3], have emerged as promising tools for tackling this challenge with polynomial overhead of computational resources. Efficient quantum simulations of chemistry systems promise breakthroughs in our knowledge for basic chemistry and revolutionize research in new materials, pharmaceuticals, and industrial catalysts.

The universal quantum simulation method [4] and the first quantum algorithm for simulating fermions [5] have laid down the fundamental block of quantum chemistry simulation. Based on these techniques and quantum phase estimation algorithm [6], Aspuru-Guzik et al. presented a quantum algorithm for preparing ground states undergoing an adiabatic evolution [7], and many theoretical and experimental works [8–24] have been developed since then. In 2002, Somma et al. proposed a scalable quantum algorithm for the simulation of molecular electron dynamics via Jordan-Wigner transformation [25]. The Jordan-Wigner transformation directly maps the fermionic occupation state of a particular atomic orbital to a state of qubits, which enables the quantum simulation of chemical systems on a quantum computer. Then, the Bravyi-Kitaev transformation [26–30] encodes both locality of occupation and parity information onto the qubits, which is more efficient in operation complexity. In 2014, Peruzzo et al. developed the variational quantum eigensolver (VQE) [18, 31], which finds a good variational approximation to the ground state of a given Hamiltonian for a particular choice of ansatz. Compared to quantum phase estimation and Trotterization of the molecular Hamiltonian, the VQE requires a lower number of controlled operations and shorter coherence time. However, the VQE is a classical and quantum hybrid algorithm; the optimizer is performed on a classical machine.

Meanwhile, implementations of quantum chemistry simulation have been developing steadily. Studies in present-day quantum computing hardware have been carried out, such as nuclear magnetic resonance system [32, 33], photonic system [34–36], nitrogen-vacancy center system [37], trapped ion [38, 39], and superconducting system [40–42]. Rapid development in quantum computer hardware with even the claims of quantum supremacy greatly stimulates the expectation of its real applications. Quantum chemistry simulation is considered a real application in Noisy Intermediate-Scale Quantum (NISQ) computers [22, 43, 44]. The FQE is an effort on this background. In the FQE, not only calculation of Hamiltonian matrix part is done on quantum computer, but also the optimization by gradient descent is performed on quantum computer. FQE can be used in near-term NISQ computers and in future fault-tolerant large quantum computers.

## 2. Method

### 2.1. Preparing the Hamiltonian for Quantum Chemistry Simulation

A molecular system contains a collection of nuclear charges *Z*_*i*_ and electrons. The fundamental task of quantum chemistry is to solve the eigenvalue problem of the molecular Hamiltonian. The eigenstates of the many-body Hamiltonian determine the dynamics of the electrons as well as the properties of the molecule. The corresponding Hamiltonian of the system includes kinetic energies of nuclei and electrons and the Coulomb potentials of nuclei-electron, nuclei-nuclei, and electron-electron, and it can be expressed in first quantization as

(1) $$\begin{array}{c} H_{\text{o}}=-\underset{i}{\sum }\frac{\nabla_{R_{i}}^{2}}{2M_{i}}-\underset{i}{\sum }\frac{\nabla_{r_{i}}^{2}}{2}-\underset{i,j}{\sum }\frac{Z_{i}}{\left|R_{i}-r_{j}\right|}+\underset{i,j>i}{\sum }\frac{Z_{i}Z_{j}}{\left|R_{i}-R_{j}\right|}+\underset{i,j>i}{\sum }\frac{1}{\left|r_{i}-r_{j}\right|}, \end{array}$$

in atomic units (*ℏ* = 1), where *R*_*i*_, *Z*_*i*_, *M*_*i*_ and *r*_*i*_ are the positions, charges, and masses of the nuclei and the positions of the electrons, respectively. Under the Born-Oppenheimer approximation which assumes the nuclei as a fixed classical point, this Hamiltonian is usually rewritten in the particle number representation in a chosen basis:

(2) $$\begin{array}{c} H=\underset{ij}{\sum }h_{ij}a_{i}^{†}a_{j}+\frac{1}{2}\underset{ijkl}{\sum }h_{ijkl}a_{i}^{†}a_{j}^{†}a_{k}a_{l}+\cdots , \end{array}$$

where ⋯ denotes higher-order interactions and *a*_*i*_^†^ and *a*_*j*_ are the creation and annihilation operators of particle in orbital *i* and *j*, respectively. The parameters *h*_*ij*_ and *h*_*ijkl*_ are the one-body and two-body integrations in the chosen basis functions {*ψ*_*i*_}. In Galerkin formulation, the scalar coefficients in Equation (2) can be calculated by

(3) $$\begin{array}{c} h_{ij}=\langle \psi_{i}\left|\left(-\frac{\nabla_{i}^{2}}{2}-\underset{\text{A}}{\sum }\;\;\frac{Z_{\text{A}}}{\left|r_{i}-R_{\text{A}}\right|}\right)\right|\psi_{j}\rangle ,\\ h_{ijkl}=\langle \psi_{i}\psi_{j}\left|\frac{1}{\left|r_{i}-r_{j}\right|}\right|\psi_{k}\psi_{l}\rangle , \end{array}$$

In order to perform calculations on a quantum computer, we need to map fermionic operators to qubit operators. We choose the Jordan-Wigner transformation to achieve this task due to its straightforward expression.

The Jordan-Wigner transformation maps Equation (2) into a qubit Hamiltonian form:

(4) $$\begin{array}{c} H=\underset{i,\alpha }{\sum }h_{\alpha }^{i}\sigma_{\alpha }^{i}+\underset{i,j,\alpha ,\beta }{\sum }h_{\alpha \beta }^{ij}\sigma_{\alpha }^{i}\sigma_{\beta }^{j}+\cdots , \end{array}$$

where Roman indices *i* and *j* denote the qubit on which the operator acts and Greek indices *α* and *β* refer to the type of Pauli operators; i.e., *σ*_*x*_^*i*^ means Pauli matrix *σ*_*x*_ acting on a qubit at site *i*. Apparently, *H* in Equation (2) is a linear combination of unitary Pauli matrices. The methods used in this paper finding the molecular ground state and its energy are all based on it.

In this work, we present the FQE to find the molecular ground-state energy by gradient descent iterations. Gradient descent is one of the most fundamental ways for optimization that looks for the target energy value along the direction of the steepest descent. Here, it is performed in a quantum computer with the help of linear combination of unitary operators. We analyze the relationships between the gradient descent iteration depth and the precision of the ground-state energy. The explicit quantum circuit to implement the algorithm is constructed. As illustrative examples, the ground-state energies and electronic structures of four molecules, H_2_, LiH, H_2_O, and NH_3_ are presented. Taking H_2_O and NH_3_ as examples, a comparison between the FQE and VQE, a representative hybrid method, is given. FQE can be accelerated further by harnessing a perturbation theory in chemical precision. Finally, we analyze the computation complexity of FQE and summarize the results.

### 2.2. Quantum Gradient Descent Iteration

The classical gradient descent algorithm is usually employed to obtain the minimum of an target function *f*(**X**). One starts from an initial point **X**^(0)^ = (*x*_1_^0^, *x*_2_^0^, ⋯, *x*_*N*_^0^) ∈ ℝ^*N*^, then moves to the next point along the direction of the gradient of the target function, namely,

(5) $$\begin{array}{c} X^{\left(t+1\right)}=X^{\left(t\right)}-\gamma_{0}\nabla f\left(X^{\left(t\right)}\right), \end{array}$$

where *γ*_0_ is a positive learning rate that determines the step size of the iteration. In searching the minimum energy of a Hamiltonian, the target function can be expressed as a quadratic optimization problem in the form *f*(**X**) = **X**^*T*^**HX**. At point **X**, the gradient operator of the objective function can be expressed as

(6) $$\begin{array}{c} \nabla f\left(X\right)=2HX. \end{array}$$

**Figure 1:**
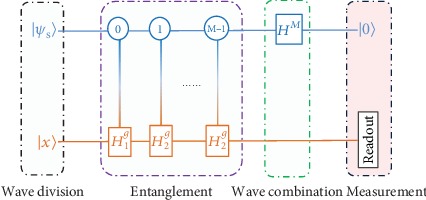
Quantum circuit for gradient descent. |**x**〉 and |*ψ*_*s*_〉 denote the initial state of the work system and ancilla syetem, respectively. The controlled operations that acted on the work system are ∑_*i*=0_^*M*−1^|*i*〉〈*i*| ⊗ *H*_*i*_^*g*^. *H*^*M*^ denotes *m* = log_2_*M* number Hadamard gates. At the end of the circuit, we measure the final state of the ancilla registers. If all ancilla qubits are |0〉, the work systerm collapses into state |**x**^(*t* + 1)^〉.

Then, the gradient descent iteration can be regarded as an evolution of **X** under operator **H**,

(7) $$\begin{array}{c} |X^{\left(t+1\right)}\rangle =|X^{\left(t\right)}\rangle -\gamma H|X^{\left(t\right)}\rangle , \end{array}$$

where *γ*_0_ is redefined as *γ* = 2*γ*_0_. In the quantum gradient descent, vector **X** is replaced by quantum state |**X**〉 = ∑_*j*_*x*_*j*_|**j**〉/‖**X**‖, where *x*_*j*_ is the *j*th elements of the vector, |**j**〉 is the *N*-dimensional computational basis, and ‖**X**‖ is the modulus of vector **X**. Denote **H**^*g*^ = **I** − *γ ***H** and it can be expressed as

(8) $$\begin{array}{c} H^{g}=\overset{M}{\underset{i=1}{\sum }}\beta_{i}H_{i}^{g}, \end{array}$$

where *M* is the number of Pauli product terms in **H**^*g*^. Then, the gradient descent process can be rewritten as

(9) $$\begin{array}{c} |X^{\left(t+1\right)}\rangle =H^{g}|X^{\left(t\right)}\rangle =\overset{M}{\underset{i=1}{\sum }}\beta_{i}H_{i}^{g}|X^{\left(t\right)}\rangle , \end{array}$$

where **H**^*g*^ is a linear combination of unitary operators (LCU) which was proposed in [45] in designing quantum algorithms and studied extensively [46–52]. This nonunitary evolution can be implemented in a unitary quantum circuit by adding ancillary qubits that transform it into unitary evolution in a larger space [53]. The realization of LCU can be viewed as a quantum computer wave function passing through *M*-slits and operated by a unitary operation in each slit, and then, the wave functions are combined and the result of the calculation is read out by a measurement [49]. As shown in Figure 1, we perform the evolution described by Equation (9) with the following four steps.

Wave division: the register is a composite system which contains a work system and an ancillary register. Firstly, the initial point **X** = (*x*_1_, ⋯,*x*_*N*_)^*T*^ is efficiently mapped as an initial state |**X**^(*t*)^〉 of the work system. In quantum chemistry, Hartree-Fock (HF) product state is usually used as an initial state. And the ancillary register is initialized from |0〉^*m*^, where *m* = log_2_*M*, to a specific superposition state |*ψ*_*s*_〉,

(10) $$\begin{array}{c} |\psi_{s}\rangle =\frac{1}{\mathbb{C}}\overset{M-1}{\underset{i=0}{\sum }}\beta_{i}|i\rangle , \end{array}$$

where $\mathbb{C}=\sqrt{\sum_{i=0}^{M-1}\beta_{i}^{2}}$ is a normalization constant and |*i*〉 is the computational basis. This is equivalent to let the state |**x**^(*t*)^〉 pass through *M*-slits. *β*_*i*_ is a factor describing the properties of the slit, which is determined by the forms of the Hamiltonian in Equation (8). This can be done by the initialization algorithm in [54]. Moreover, the quantum random access memory (qRAM) approach can be used to prepare |**x**^(*t*)^〉 and |*ψ*_*s*_〉, which consume *O*(log*N*) and *O*(log*M*) basic steps or gates, respectively, after qRAM cell is established. We denote the whole state of the composite system as |*Φ*〉 = |*ψ*_*s*_〉|**x**^(*t*)^〉.

Entanglement: then, a series of ancillary system-controlled operations ∑_*i*=0_^*M*−1^|*i*〉〈*i*| ⊗ *H*_*i*_^*g*^ are implemented on the work qubits. The work qubits and the ancilla register are now entangled, and the state is transformed into

(11) $$\begin{array}{c} |\Phi \rangle ⟶\frac{1}{\mathbb{C}}\left(\overset{M-1}{\underset{i=0}{\sum }}\beta_{i}|i\rangle H_{i}^{g}|x^{\left(t\right)}\rangle \right). \end{array}$$

The corresponding physical picture is that different unitary operations are implemented simultaneously in different subspaces, corresponding to different slits.

Wave combination: we perform *m* Hadamad gates on ancillary register to combine all the wave functions from the *M* different subspaces. We merely focus on the component in a subspace where the ancillary system is in state |0〉. The state of the whole system in this subspace is

(12) $$\begin{array}{c} |\Phi_{0}\rangle ⟶\frac{1}{\mathbb{C}\sqrt{2^{m}}}\left(|0\rangle \overset{M-1}{\underset{i=0}{\sum }}\beta_{i}H_{i}^{g}|x^{\left(t\right)}\rangle \right). \end{array}$$

Measurement: then, we measure the ancillary register. If we obtain |0〉, our algorithm succeeds and we obtain the state $\left(1/\mathbb{C}\sqrt{2^{m}}\right)\left(|0\rangle \sum_{i=0}^{M-1}\beta_{i}H_{i}^{g}|x^{\left(t\right)}\rangle \right)$, where the work system is in |**x**^(*t* + 1)^〉 = **H**^*g*^|**x**^(*t*)^〉. And then, this will be used as input for the next iteration in the quantum gradient descent process. The probability of obtaining |0〉 for the state is

(13) $$\begin{array}{c} P_{s}={\left\|H^{g}|x^{\left(t\right)}\rangle \right\|}^{2}/{\mathbb{C}}^{2}M. \end{array}$$

The successful probability after *n* measurements is 1 − (1 − ‖**H**^*g*^|**x**^(*t*)^〉‖^2^/*ℂ*^2^*M*)^*n*^, which is an exponential function of *n*. The number of measurements is *ℂ*^2^*M*/‖**H**^*g*^|**x**^(*t*)^〉‖^2^. The measurement complexity will grow exponentially with respect to the number of iteration steps [55]. Alternatively, one can use the oblivious amplitude amplification [51] to amplify the amplitude of the desired term (ancillary qubits in state |0〉) up to a deterministic order with $O\left(\sqrt{M}\right)$ repetitions before the measurement. Then, the measurement complexity will be the product of iteration depth *k* and $O\left(\sqrt{M}\right)$, linearly dependent on the number of iteration steps. After obtaining |0〉, we can continue the gradient descent process by repeating the above four steps, with |**x**^(*t*)^〉 replaced by |**x**^(*t* + 1)^〉 in a wave-division step. We can preset a threshold defined as *ε* = |〈**x**_*t*_ | *H* | **x**_*t*_〉 − 〈**x**_*t*+1_ | *H* | **x**_*t*+1_〉|/〈**x**_*t*_ | *H* | **x**_*t*_〉 as criterion for stopping the iteration. Thus, we judge if the iterated state satisfies the criterion by measuring the expectation value of Hamiltonian around the expected number of iteration, which is easier than constructing the tomography. If the next iterative state |**x**^(*t* + 1)^〉 does not hit our preset threshold, this output |**x**^(*t* + 1)^〉 will be regarded as the new input state |**x**^(*t*)^〉 and run the next iteration. Otherwise, the iteration can be terminated and the state |**x**^(*t* + 1)^〉 is the final result |**x**_*f*_〉, as one good approximation of the ground state. The ground-state energy can be calculated by 〈**x**_*f*_ | *H* | **x**_*f*_〉.

Measuring the expectation values during the iteration procedure will destroy the state of the work system, stopping the quantum gradient descent process. So, determining the iteration depth *k* in advance is essential. After *k* times iterations, the approximation error is limited to (ignoring constants)

(14) $$\begin{array}{c} \varepsilon \leq O\left({\left(\frac{1-\gamma \lambda_{2}}{1-\gamma \lambda_{1}}\right)}^{k}N\right), \end{array}$$

where *λ*_1_ and *λ*_2_ are the two largest absolute values of the eigenvalues of Hamiltonian **H** (see Supplemental Material ([Supplementary-material supplementary-material-1]) for proof). The iteration depth

(15) $$\begin{array}{c} k=O\left(\log\frac{N}{\varepsilon }\right) \end{array}$$

is logarithmically dependent on the system size and the inverse of precision. The algorithm may be terminated at a point with a preset precision *ε*. It can be seen that the choise of *γ* has little impact on converge rate when *γ* is large. This makes this algorithm very robust to this parameter. The rate of convergence primarily depends upon the ratio of *λ*_1_ and *λ*_2_. The gap between the iterative result and the ground state depends on the choice of initial point. If we choose an ansatz state with a large overlap with the exact ground state, the iterative process will converge to the ground state in fewer iterations. Usually, the mean-field state which represents a good classical approximation to the ground state of Hamiltonian H, such as a Hartree-Fock (HF) product state, is chosen as an initial state. Compared to the VQE, the FQE does not need to make measurements of the expectation values of Hamiltonian during each iteration procedure and this substantially reduces the computation resources.

### 2.3. Perturbation Theory

The FQE involves multitime iterations to obtain an accurate result, which is difficult to implement in the present-day quantum computer hardware. Here, we present an approximate method to find the ground state and its energy by using the gradient descent algorithm and perturbation theory. The perturbation theory is widely used and plays an important role in describing real quantum systems, because it is impossible to find exact solutions to the Schrodinger equation for Hamiltonians even with moderate complexity. The Hamiltonian described by Equation (4) can be divided into two classes, **H**_0_ and **H**′. **H**_0_ consists of a set of Pauli terms containing only *σ*_*α*=*z*_^*i*^ and the identity matrices, and Pauli terms *σ*_*α*=*x*,*y*_^*i*^ belong to **H**′. **H**_0_ is a diagonal matrix with exact solutions that can be regarded as a simple system. **H**′ usually is smaller compared to **H**_0_ and is treated as a “perturbing” Hamiltonian. The energy levels and eigenstates associated with the perturbed system can be expressed as “corrections” to those of the unperturbed system. We begin with the time-independent Schrodinger equation:

(16) $$\begin{array}{c} H|\psi_{n}\rangle =\left(H_{0}+H^{\prime }\right)|\psi_{n}\rangle =E_{n}|\psi_{n}\rangle , \end{array}$$

where *E*_*n*_ and |*ψ*_*n*_〉 are the *n*th energy and eigenstate, respectively. Unperturbed Hamiltonian **H**_0_ satisfies the time-independent Schrodinger equation: **H**_0_|*n*〉 = *E*_*n*_^(0)^|*n*〉. Our goal is to express *E*_*n*_ and |*ψ*_*n*_〉 in terms of *E*_*n*_^0^ and |*n*〉. Denote the expectation value of **H**′ as **H**′_*nn*_ = 〈*n* | **H**′ | *n*〉, and it is easy to see that 〈*n* | **H**′ | *n*〉 is zero because **H**′ only contains Pauli terms *σ*_*α*=*x*,*y*_^*i*^. In the first-order approximation, the energies and eigenstates are expressed as

(17) $$\begin{array}{c} E_{n}=E_{n}^{\left(0\right)},\\ |\psi_{n}\rangle =|n\rangle -\underset{m\neq n}{\sum }\frac{H_{mn}^{\prime }}{E_{m}^{\left(0\right)}-E_{n}^{\left(0\right)}}|m\rangle . \end{array}$$

In the second-order approximation, they are

(18) $$\begin{array}{c} E_{n}=E_{n}^{\left(0\right)}+\underset{m\neq n}{\sum }\frac{{\left|H_{mn}^{\prime }\right|}^{2}}{E_{m}^{\left(0\right)}-E_{n}^{\left(0\right)}},\\ |\psi_{n}\rangle =|n\rangle -\underset{m\neq n}{\sum }\frac{H_{mn}^{\prime }}{E_{m}^{\left(0\right)}-E_{n}^{\left(0\right)}}|m\rangle -\frac{1}{2}\underset{m\neq n}{\sum }\frac{{\left|H_{mn}^{\prime }\right|}^{2}}{{\left(E_{m}^{\left(0\right)}-E_{n}^{\left(0\right)}\right)}^{2}}|n\rangle ,+\underset{m\neq n}{\sum }\left[\underset{k\neq n}{\sum }\frac{H_{mn}^{\prime }H_{kn}^{\prime }}{\left(E_{m}^{\left(0\right)}-E_{n}^{\left(0\right)}\right)\left(E_{k}^{\left(0\right)}-E_{n}^{\left(0\right)}\right)}\right]|m\rangle . \end{array}$$

The matrix elements in the first- and second-order approximations can be obtained by one iteration of the quantum circuit in Figure 1. Here, we let **H**′ be equal to **H**^*g*^. Explicitly, the first-order approximation only involves **H**′_*mn*_, a series of transition probabilities of the state after **H**′ implemented on state |*ni*, and they can be obtained by performing the quantum circuit of Figure 1 directly. For the second-order approximation, matrix elements such as value |**H**′_*mn*_|^2^ and **H**′_*mn*_**H**′_*kn*_ can be calculated by **H**′_*mn*_. Then, the approximate ground energy and ground state up to the second order are obtained. We will show the performance of the FQE and perturbation theory in the next section.

## 3. Results

### 3.1. Calculations of Four Molecules

**Figure 2:**
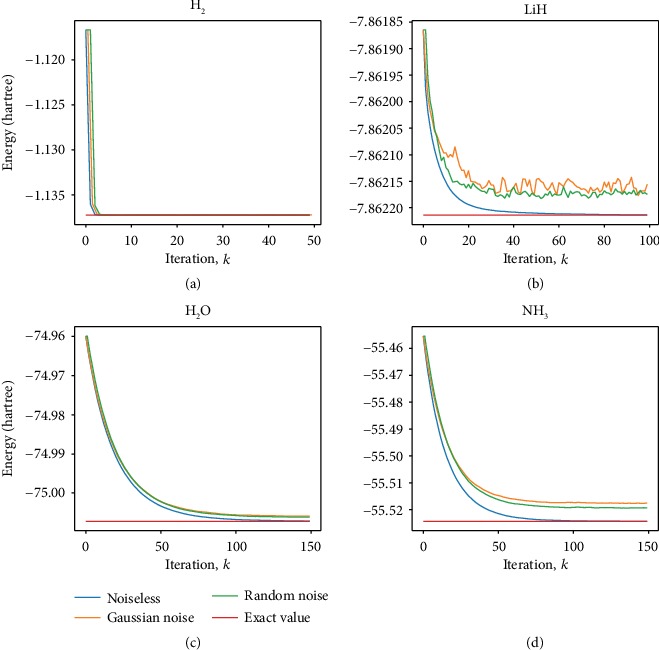
(a), (b), (c), and (d) show the convergence to ground-state energies by the FQE for H_2_, LiH, H_2_O, and NH_3_ molecules, respectively. The numerical simulations are carried out with fixed interatomic distance. The exact value corresponding to Hamiltonian diagonalization energy (red line). The initial state is chosen as the Hartree-Fock product state in all four cases. The final values of the lines for exact ground-state energy (red line) and for the three iteration results, noiseless case (blue line), random noisy case (green line), and Gaussian noisy case (orange line).

**Figure 3:**
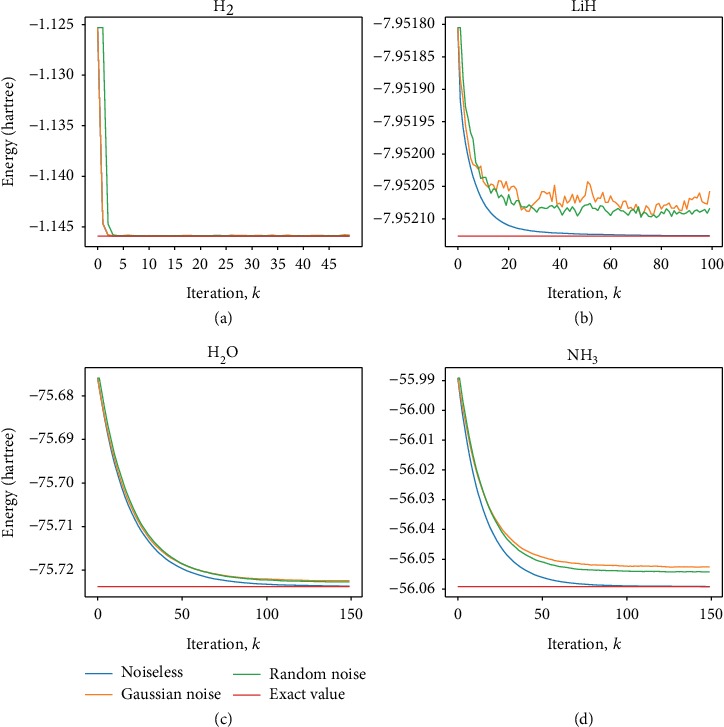
(a), (b), (c), and (d) show the gradient descent iteration process for convergence of ground-state energy of H_2_, LiH, H_2_O, and NH_3,_ respectively. The qubit Hamiltonians of the four molecules are obtained by STO-6G basis, which is more accurate than STO-3G basis.

To demonstrate the feasibility of this FQE with gradient descent iteration, we carried out calculations on the ground-state energy of H_2_, LiH diatomic molecules, and two relatively complex molecules H_2_O and NH_3_. We used a common molecular basis set, the minimal STO-3G basis. Via the Jordan-Wigner transformation, the qubit Hamiltonians of these molecules are obtained. The Hamiltonians of H_2_, LiH, H_2_O, and NH_3_ contain 15, 118, 252, and 3382 Pauli matrix product terms, respectively. The dimensions of the Hamiltonians of H_2_, LiH, H_2_O, and NH_3_ are 16, 64, 4096, and 16384, respectively, which correspond to 4, 6, 12, and 14 numbers of qubits, respectively. In all four simulations, the work system was initialized to the HF state |**x**_*h*_〉 and the learning rate is chosen as *γ* = 1. As shown in Figure 2, after about 120 iterations, the molecular energy of H_2_O converges to -74.94 a.u., only 0.0013346% discrepancy with respect to the exact value of -74.93 a.u. obtained via Hamiltonian diagonalization. The NH_3_ calculation yields (-55.525 a.u.) after 80 iterations matched very well with the diagonalization (-55.526 a.u.). For the study of atomic molecular structures and chemical reactions, these results are sufficiently accurate. For more complex basis set STO-6G, the results are about the same (see Figure 3), and the details are given in Supplemental Material. The converge rates of the four molecules depend on the system size and the ratio of the two largest absolute eigenvalues of the Hamiltonian **H**, which are consistent with the theoretical analysis above.

**Figure 4:**
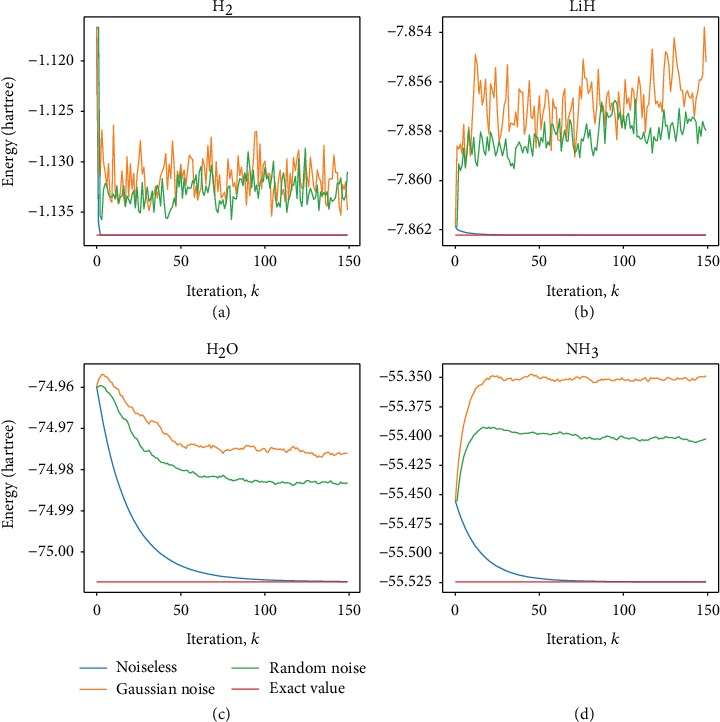
Influence of large noise on the FQE in (a) H_2_, (b) LiH, (c) H_2_O, and (d) NH_3_ molecules, respectively. The amplitude of the random noise is 0.1, and the Gaussian noise parameters are *μ* = 0 and *σ* = 0.1/3.

We also studied the influence of noises which is also shown in Figure 2. The noise term is chosen in the form of ∑_*i*=1_^*N*^*δα*_*i*_*σ*_*z*_ added to the Hamiltonian to simulate decoherence. Then, we add a term $|\delta \vec{x}\rangle$ on the iterative state |**x**^*k*^〉 to simulate measurement error and renormalize the iterative state as $|x^{\left(k\right)}\rangle ⟶\left(|x^{\left(k\right)}\rangle +|\delta \vec{x}\rangle \right)/\left\||x^{\left(k\right)}\rangle +|\delta \vec{x}\rangle \right\|$. We set a random noise (amplitude 0.01) and a Gaussian noise (*μ* = 0, *σ* = 0.01/3) for H_2_ and LiH. For H_2_O and NH_3_, we choose a random noise (amplitude 0.02) and a Gaussian noise (*μ* = 0, *σ* = 0.02/3). The results still converge to the exact values in chemical precision (1.6 × 10 − 3 a.u). This indicates that our method is robust to a certain type of noise, which is important in the implementation of quantum simulation on near-term quantum devices. For more noisy situations, see Supplemental Material for details, where the parameters of noise are 10 times the above values (see Figure 4). The convergence deteriorates and some oscillations occur as the number of iterations increases.

**Figure 5:**
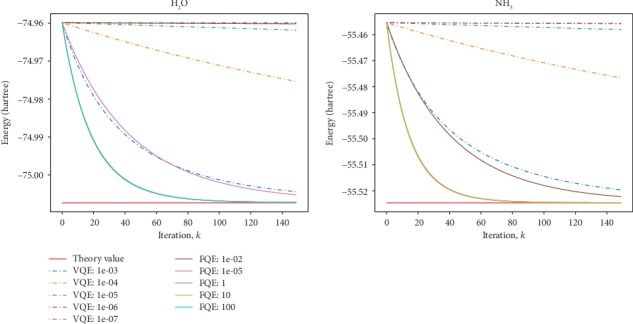
The exact comparison of the FQE and VQE for searching ground-state energy of H_2_O and NH_3_ molecules, respectively. The red color lines labeled as “Theory value” are the exact values of ground-state energy. The labels of the right symbols denote different learning rates.

In Figure 5, a comparison with the VQE is shown for H_2_O and NH_3_. In VQE calculation, the initial state |**x**_0_〉 is mapped to an ansatz state by a parameterized unitary operation $|x\left(\vec{\theta }\right)\rangle =U\left(\vec{\theta }\right)|x_{0}\rangle$. The VQE solves for the parameter vector $\vec{\theta }$ with a classical optimization routine. Here, we adopt the standard gradient descent method as the classical optimizer in the VQE. The parameter is updated by $\vec{\theta }⟶\vec{\theta }-\gamma \left(\left(f\left(\vec{\theta }+\Delta \vec{\theta }\right)-f\left(\vec{\theta }\right)\right)/\Delta \vec{\theta }\right)$. We performed numerical simulations of the VQE for the two molecules. When the learning rate *γ* ≥ 10^−3^, the VQE does not converge to the ground state. So, in order to compare with each other, we choose the proper learning rates in two methods separately. In both cases, the initial ansatz state is prepared as the HF product state. In H_2_O and NH_3_, the VQE converges the fastest with the learning rate *γ* = 10^−3^. The FQE converges faster with larger and larger learning rate until a fixed speed is reached. As shown in Figure 5, the FQE generally converges faster than the VQE and the advantage will be more obvious in complex molecules.

**Figure 6:**
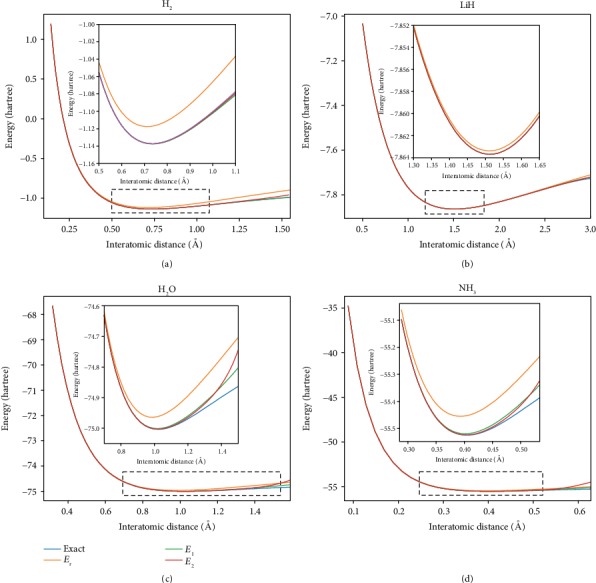
Theory results (blue lines) and zero-order (orange lines), first-order (green lines), and second-order (red lines) energy plots of outcomes from numerical simulations, for several interatomic distances for H_2_, LiH, H_2_O (between the oxygen atom and one hydrogen atom), and NH_3_ (between the nitrogen atom and the plane formed by the three hydrogen atoms).

The above examples are calculated in fixed interatomic distance of the molecules. If we want to calculate the interatomic distance corresponding to the most stable structure, the variation of interatomic distances is necessary. In Figure 6, four examples are given to illustrate the performance of the perturbation theory. To obtain the potential-energy surfaces for H_2_, LiH, H_2_O, and NH_3_ molecules, we studied the dependence of ground-state energy of their molecules on the variating interatomic distances, between the two atoms in H_2_ and LiH, the distance between the oxygen atom and one hydrogen atom (the two hydrogen atoms are symmetric with respect to the oxygen atom) in H_2_O, and the distance between the nitrogen atom and the plane formed by the three hydrogen atoms in NH_3_. The lowest energy in potential-energy surfaces corresponds to the most stable structure of the molecules. As shown in the picture, the ground-state energy of each molecule calculated under the second-order approximation is already quite close to its exact value, which is obtained from Hamiltonian diagonalizations. The energy values up to the second-order correction are compared with their exact values at the most stable interatomic distance corresponding to the lowest energy in Table 1. It can be seen that the second-order approximation has already given results in chemical precision.

### 3.2. Analysis of Computational Complexity

Here, we analyze the complexity of our algorithm. Usually, a quantum algorithm complexity involves two aspects: qubit resources and gate complexity. For qubit resources, the number of ancilla qubits is log*M*, where *M* is the number of Pauli terms in qubit from the Hamiltonian. For gate complexity, the “Wave division” part needs *𝒪*(log*N* + log*M*) basic steps for state preparation. The dominant factor is the number of controlled operations in the “Entanglement” part in Figure 1. Controlled **H**_*i*_^*g*^ can be decomposed into *𝒪*(*M*log*M*log*N*) basic gates [56, 57]. The “Wave combination” part just comprises log*M* Hadamard gates. Totally, the FQE requires in each iteration about *𝒪*(*M*log*M*log*N*) basic gates for implementation. If the wave function is expressed by *O*(*N*) Gaussian orbitals, fermion Hamiltonians contain *O*(*N*^4^) second-quantized terms; consequently, the qubit Hamiltonians have *M* = *O*(*N*^4^) Pauli terms. The qubit resource and gate complexity can be reduced to *O*(*N*) and *O*(*N*^4^), respectively. In some applications, the perturbation theory only requires one iteration, and an approximate result in chemical precision can be obtained.

## 4. Summary

An efficient quantum algorithm, full quantum eigensolver (FQE), for calculating the ground-state wave function and the ground energy using gradient descent (FQE) was proposed, and numerical simulations are performed for four molecules. In the FQE, the complexity of basic gate operations is polylogarithmical to the number of single-electron atomic orbitals. It achieves an exponential speedup compared with its classical counterparts. It has been shown that the FQE is robust against noises of reasonable strengths. For very noisy situations that do not allow many iterations, the FQE can be combined with the perturbation theory that gives the ground state and its energy in chemical precision with one-time iteration. The FQE is exceptionally useful in quantum chemistry simulation, especially for the near-term NISQ applications. The FQE is a full quantum algorithm, not only applicable for NISQ computers but also directly applicable for future large-scale fault-tolerant quantum computers.

## Figures and Tables

**Table 1:** Energy values calculated by a perturbation method and the exact values in the most stable distance corresponding to the lowest ground energy.

Energy value (au)	Exact value	Zero-order value	First-order value	Second-order value
Distance (Å)				
H_2_ (0.7314)	-1.1373	-1.1171	-1.1372	-1.1372
LiH (1.5065)	-7.8637	-7.8634	-7.8637	-7.8637
H_2_O (1.0812)	-75.0038	-74.9622	75.0013	75.0032
NH_3_ (0.4033)	-55.5247	-55.4530	-55.5193	-55.5237

## Data Availability

The data that support the findings of this study are available from the corresponding authors on reasonable request.

## Supplementary Materials

Supplementary Materials Section A: error estimation and iteration complexity of FQE. Section B: FQE with STO-6G basis as input. Section C: performance of FQE with large noise. Figure S1: the gradient descent iteration process for convergence of ground-state energy of H_2_, LiH, H_2_O, and NH_3_, respectively, with STO-6G basis. Figure S2: influence of large noise on FQE in H_2_, LiH, H_2_O, and NH_3_ molecules, respectively [58].
