# Supplementary figures and images for: A Full Quantum Eigensolver for Quantum Chemistry Simulations
Source: Research (Wash D C). 2020 Mar 23;2020:1486935. doi: 10.34133/2020/1486935 (PMC7125455; doi:10.34133/2020/1486935)

$H_2$ 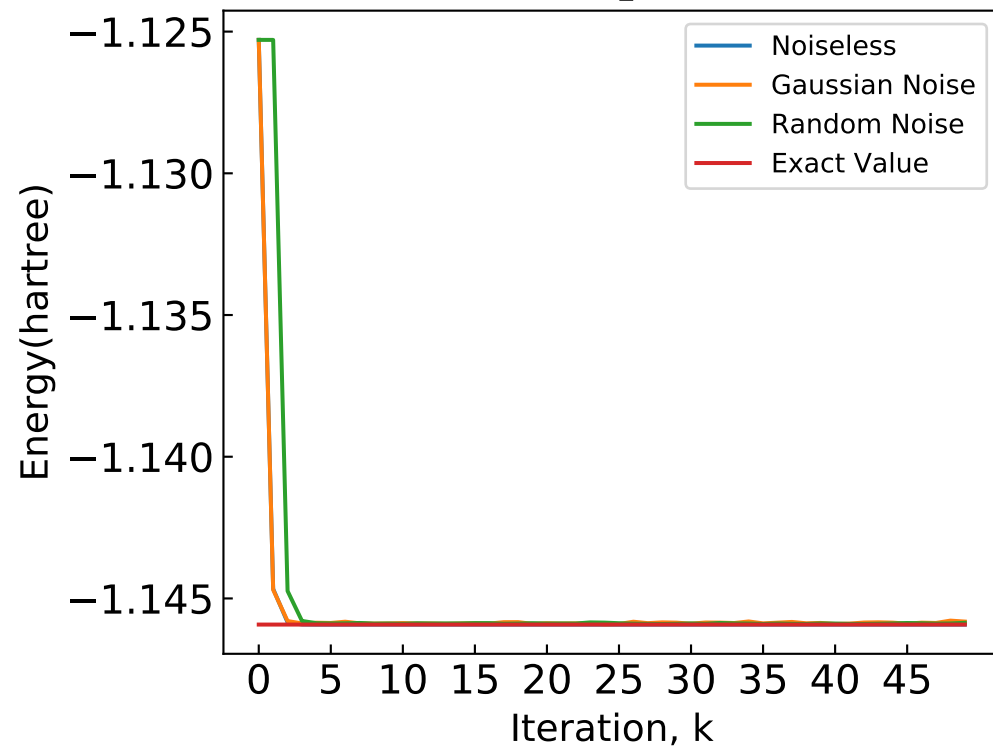 $LiH$ 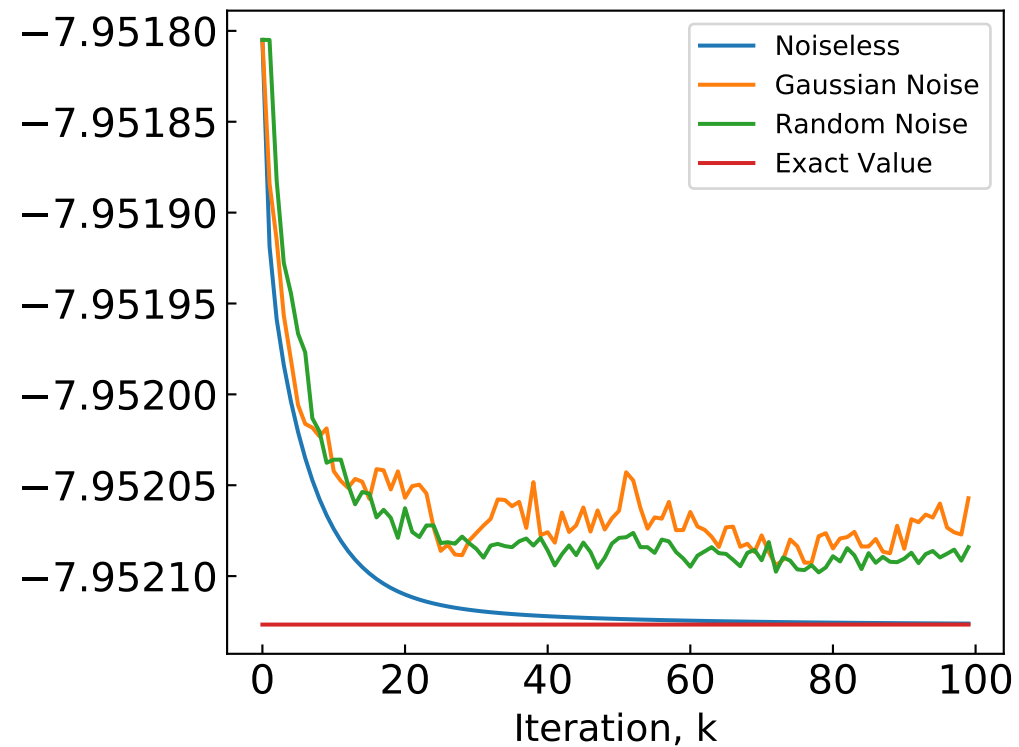 $H_2O$ 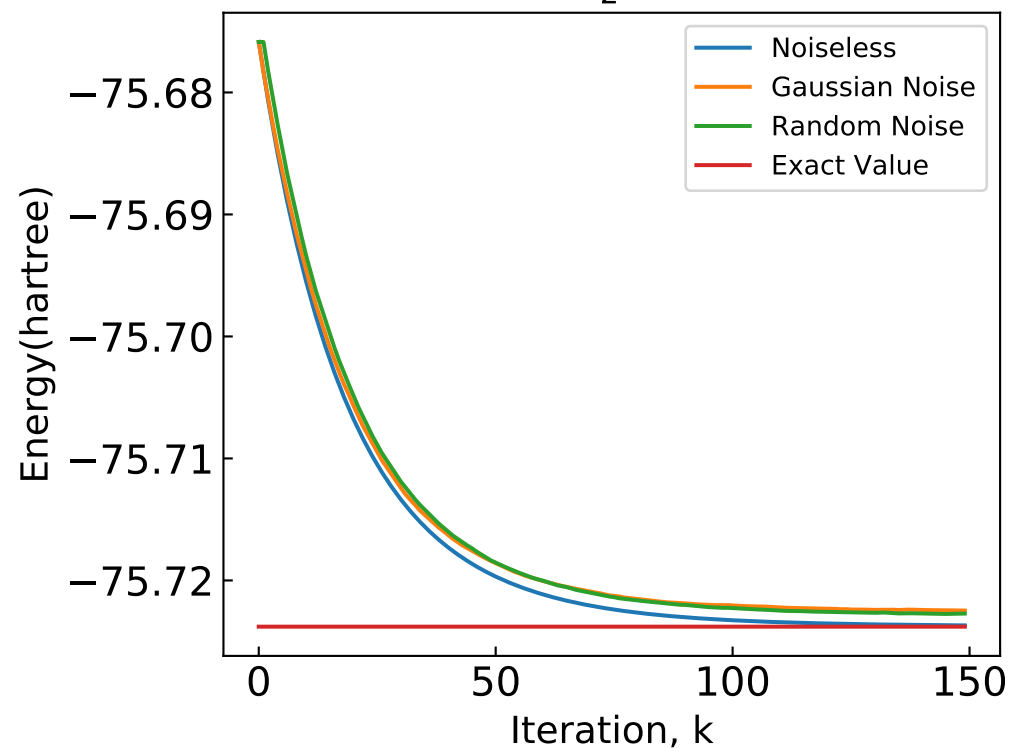 $NH_3$ 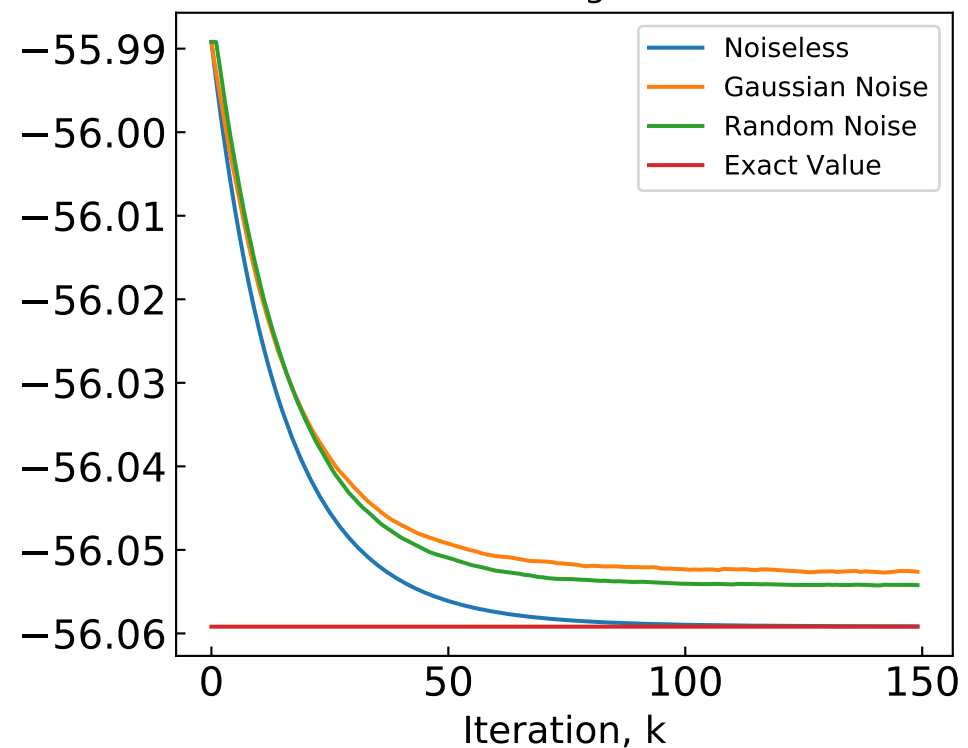

Supplement: Supplementary Materials — Section A: error estimation and iteration complexity of FQE. Section B: FQE with STO-6G basis as input. Section C: performance of FQE with large noise. Figure S1: the gradient descent iteration process for convergence of ground-state energy of H2, LiH, H2O, and NH3, respectively, with STO-6G basis. Figure S2: influence of large noise on FQE in H2, LiH, H2O, and NH3 molecules, respectively [58]. [file 1486935.f1.zip › SM1.pdf]

$H_2$ 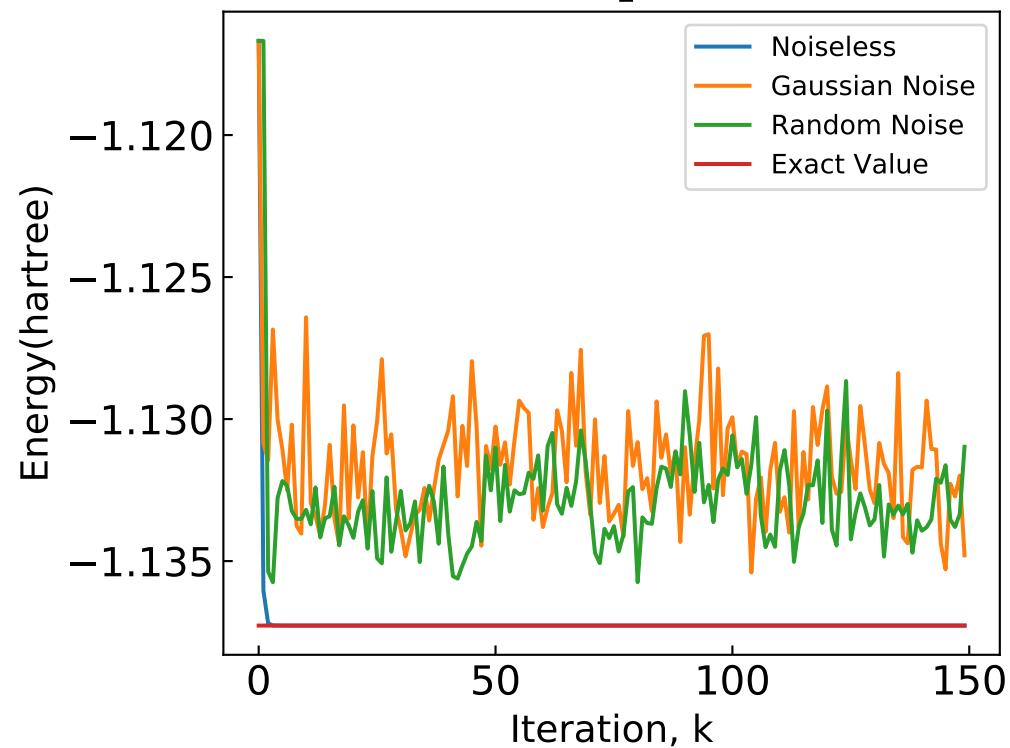 $LiH$ 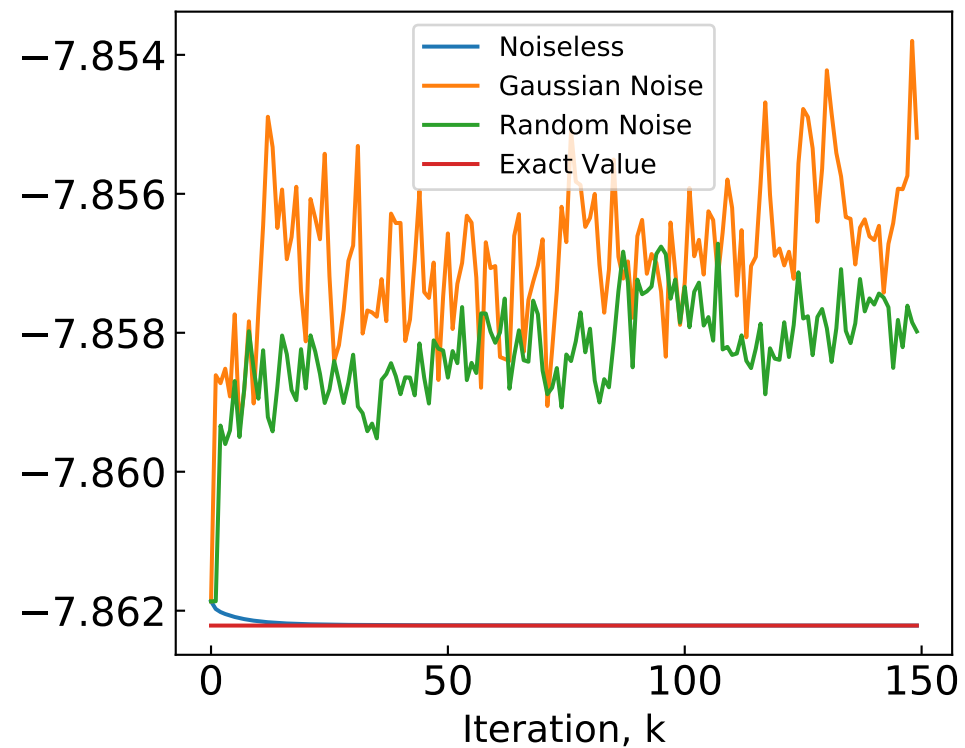 $H_2O$ 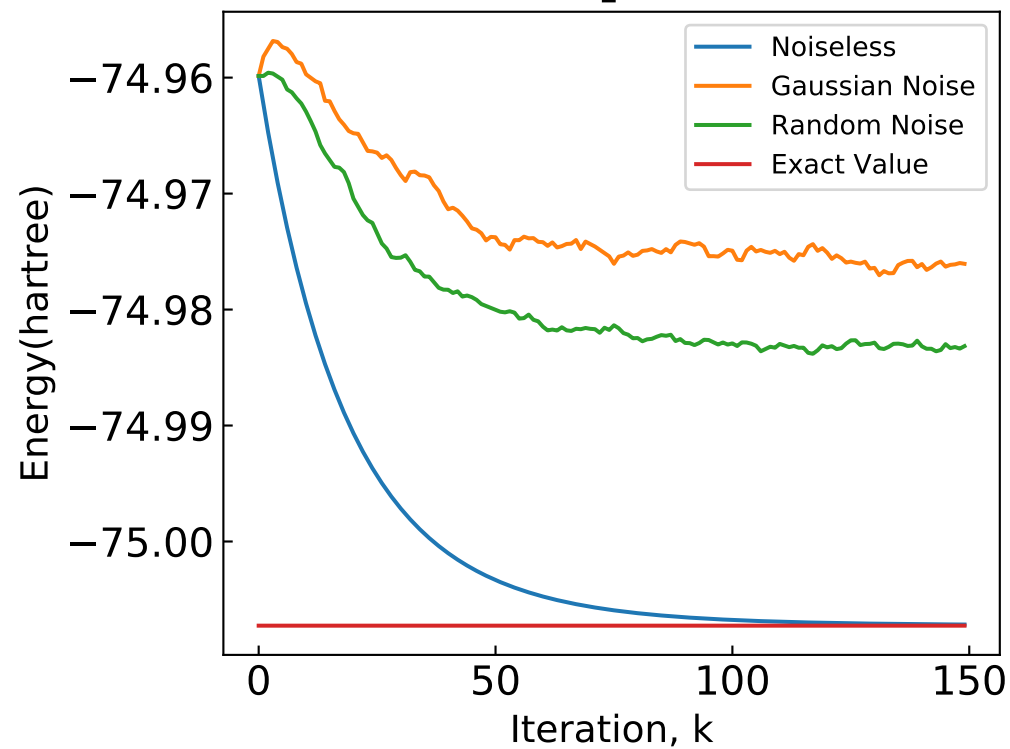 $NH_3$ 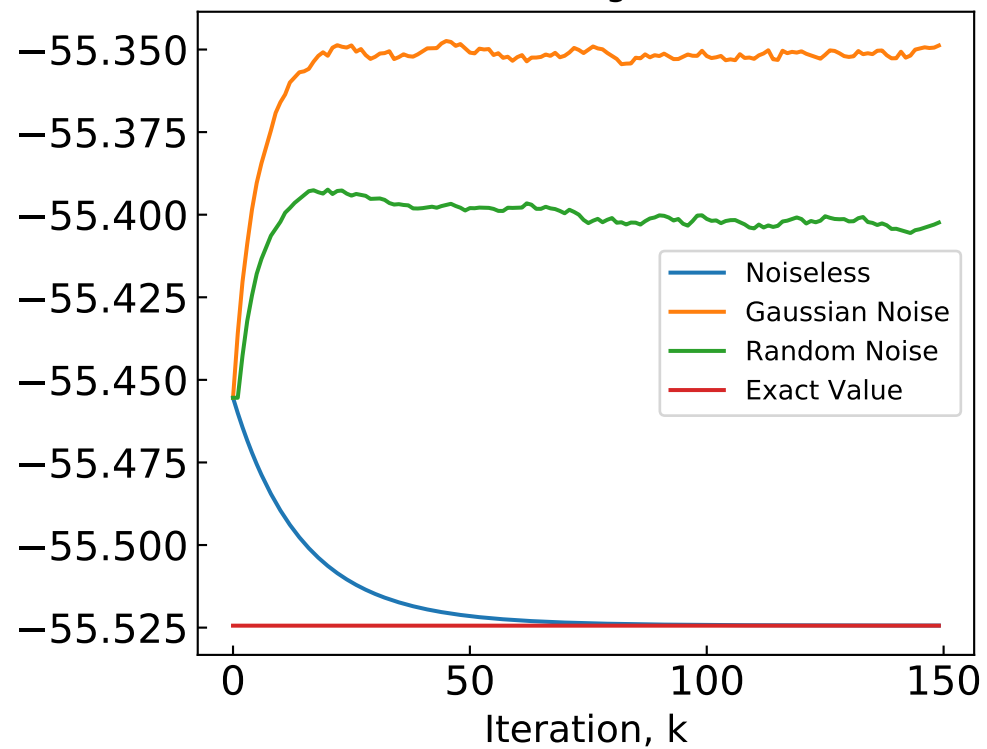

Supplement: Supplementary Materials — Section A: error estimation and iteration complexity of FQE. Section B: FQE with STO-6G basis as input. Section C: performance of FQE with large noise. Figure S1: the gradient descent iteration process for convergence of ground-state energy of H2, LiH, H2O, and NH3, respectively, with STO-6G basis. Figure S2: influence of large noise on FQE in H2, LiH, H2O, and NH3 molecules, respectively [58]. [file 1486935.f1.zip › SM2.pdf]
